# Environmental factors influencing the abundance of four species of threatened mammals in degraded habitats in the eastern Brazilian Amazon

**DOI:** 10.1371/journal.pone.0229459

**Published:** 2020-02-26

**Authors:** Juliana Teixeira-Santos, Ana Carolina da Cunha Ribeiro, Øystein Wiig, Nelson Silva Pinto, Lorrane Gabrielle Cantanhêde, Leonardo Sena, Ana Cristina Mendes-Oliveira

**Affiliations:** 1 Institute of Biological Science, Federal University of Pará, Belém, Pará, Brazil; 2 Natural History Museum, University of Oslo, Oslo, Norway; 3 Centro Universitário UniAraguaia, Goiânia, Goiás, Brazil; Texas Tech University, UNITED STATES

## Abstract

On the latest 60 years the degradation and fragmentation of native habitats have been modifying the landscape in the eastern Brazilian Amazon. The adaptive plasticity of an organism has been crucial for its long-term survival and success in these novel ecosystems. In this study, we investigated the response of four endangered species of large terrestrial mammals to the variations in the quality of their original habitats, in a context of high anthropogenic pressure. The distribution of the *Myrmecophaga tridactyla* (Giant anteater), *Priodontes maximus* (Giant armadillo), *Tapirus terrestris* (Lowland tapir) and *Tayassu pecari* (White-lipped peccary) in all sampled habitats suggests their tolerance to degradation. However, the survival ability of each species in the different habitats was not the same. Among the four species, *T*. *pecari* seems to be the one with the least ability to survive in more altered environments. The positive influence of the anthropogenically altered habitats on abundances of three of the four species studied, as observed at the regeneration areas, can be considered as a potential indication of the ecological trap phenomenon. This study reinforces the importance of the forest remnants for the survival of endangered mammal species, in regions of high anthropogenic pressure, as in the eastern Brazilian Amazon.

## Introduction

Since the 1960s the Brazilian Amazon rainforest has been degraded at a fast pace. Land use changes have led to an accumulated deforestation of 21% of this Biome by 2018 [1]. About 90% of this deforestation is concentrated in the "Deforestation Arc" [2], located in the eastern and southern portion of the area, which encompass the agricultural and cattle frontier of the Amazon rainforest in Brazil. In addition to the substitution of the forest for agriculture and pasture [3], there is also a removal of forest and soil for mining activities [4], and degradation of the forest through selective-logging [5]. All these anthropogenic processes lead to an expansion of urban and industrial infrastructure areas [6]. The consequences are changes in the dynamics of the Amazon ecosystem, reducing environmental complexity, modifying ecosystem functions and drastically impacting the regional biodiversity [7, 8, 9, 10].

The response of the fauna to the new environmental conditions may vary according to the taxon and the intensity of the anthropogenic impact. The adaptive fitness of a species is closely related to its evolutionary history. The organisms evolved based on environmental factors that shaped preferences and ecological demands over a sufficient evolutionary time to allow genotypic and phenotypic adaptations that favored and increased the fitness of the species [11]. However, rapid human-induced environmental changes (HIREC) [12] has resulted in a new reality in tropical forests, with the emergence of "novel ecosystems" that differ in composition, function and/or appearance from the past systems [13]. The response of the fauna to this phenomenon, usually associated with climate changes or invasive species, has been referred to as the "Ecological Trap"[13]. This term defines the choice or preference of an organism for a resource or habitat different from the original, even if this means reducing its fitness [13, 14].

Currently, the Amazon rainforest is not exempt from the phenomena of ecological novelties or ecological traps [13]. The fragmentation and degradation of native habitats have modified the landscape in the eastern Brazilian Amazon, with the formation of remnants of mature forests at different levels of degradation, mixed with secondary forests at different levels of regeneration and economically productive open areas [15]. The adaptive plasticity of an organism, which is its ability to suit these new environments, will be crucial for its long-term survival and success [11]. However, the survival ability of a species may be more efficient when the taxon has already been exposed to similar situations in its evolutionary past [13]. In addition, the intensity and time scale of environmental and structural changes may also interfere with these responses [16].

Mammals represent a group which is greatly threatened by environmental changes in the Amazon [8, 10]. Thirty-five species of mammals that occur in the Brazilian Amazon are listed in the Brazilian Red List of threatened species [17]. In this study, we selected four of these threatened species to study and understand factors that have influenced their abundance in a context of high anthropogenic pressure: *Myrmecophaga tridactyla* (Giant anteater), *Priodontes maximus* (Giant armadillo), *Tapirus terrestris* (Lowland tapir) and *Tayassu pecari* (White-lipped peccary). All are large neotropical mammals, which originally had a wide distribution in South America but are now considered threatened mainly by hunting and degradation of their natural habitats [17, 18].

All four target species of this study represent ancient evolutionary histories in the American continent [19, 20, 21]. Molecular analyses indicate for example that the order Xenarthra, which includes the species *M*. *tridactyla* and *P*. *maximus*, had a common origin to the order Afrotheria at the end of the Cretaceous (106 million years ago), when Africa, South America, Antarctica, and Australia still formed the Gondwana supercontinent [21]. As Xenarthrans, other representatives of terrestrial mammals, including Arctidactyla and Perisssodactyla ancestors, developed up to the Pliocene in total isolation from the rest of the placentarians [21]. During this geological period, the mammalian fauna of this continent developed morphological, physiological and behavioral adaptations making them capable of colonizing the niches developing in this region [19, 20]. These animals are therefore genuinely neotropical and, although they are widely distributed in South America, the way they use native habitats today is closely related to their evolutionary history [13].

In this study, we investigated the response of the abundance of *M*. *tridactyla*, *P*. *maximus*, *T*. *terrestris* and *T*. *pecari* to variations in the quality of their original habitats, in the eastern Brazilian Amazon. We hypothesize that environmental differences caused by anthropogenic factors alter the ability of species to tolerate and remain in a particular habitat. Based on the evolutionary history and the current ecological characteristics of the species, we discuss their ability to survive in the novelty habitats. Finally, we discuss the implications of these results for species conservation on the theoretical view of "Ecological Novelty" and "Ecological Trap" [13].

## Material and methods

### Study area

**Fig 1:**
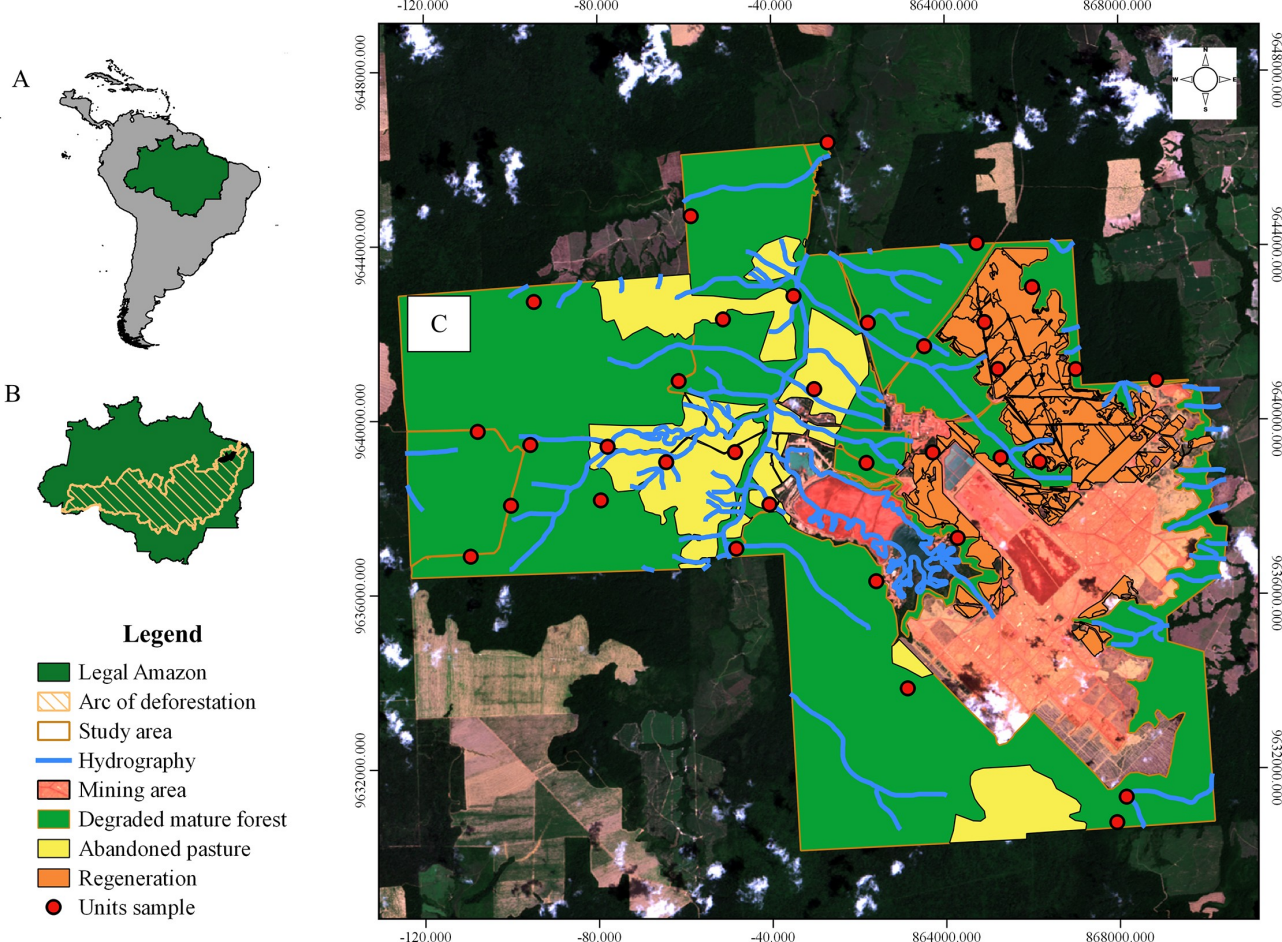
Location of the study area. (A) South America highlighting the Brazilian Amazon; (B) The deforestation Arc in hatch and the Paragominas municipality in red; (C) Limits of the study area and the spatial distribution of the 35 sampling points (camera traps) in the different habitats. Reprinted from [28] [29] under a CC BY license, with permission from [IMAZON and EOS], original copyright [2013].

The study was carried out in the area of the bauxite mine of Hydro Paragominas Company located in the Paragominas municipality, state of Pará, in the eastern Brazilian Amazon (coordinates, 3°15’14” S, 47°43’18” W) (Fig 1). According to the Köppen-Geiger classification, the climate in the area is moist tropical [22]. The original vegetation of the area was composed mostly by typical Dense Amazon Rainforest [23], with a continuous canopy ranging from 25–30 m in height, with a low dense understory and an average basal area of 20–30 m^2^/ha [24].

However, the study area region has been undergoing an intense process of forest degradation and deforestation, mainly between the 1970s and 2000s [2]. Illegal and predatory logging impoverished the region's forests, and later agro-industry and livestock farming have caused high rates of deforestation. According to the Brazilian National Institute of Space Research (INPE), in 2015 about 45% of the forest area of Paragominas had already been deforested [1] and about 60% of the forest remnants had already suffered some kind of anthropogenic impact.

The region of Paragominas also presents a high concentration of bauxite (sedimentary rock with high aluminum content), which covers about 58% of the district's soil. Bauxite is the basis for the production of aluminum. The environmental consequences of this mining activity include changes in the landscape due to the total withdrawal of vegetation as well as the removal of the fertile soil and its content of seeds, causing a decrease in local biodiversity [4, 25, 26]. In the study area, the bauxite mined areas are later reforested with native species, through the Degraded Area Rehabilitation Plan (PRAD) implemented by the Hydro Paragominas Company [27].

Anthropogenic activities have transformed the landscape of the study region into a mosaic of emerging habitats at different levels of degradation. The area cover a total of 18,764 ha, which includes: degraded mature forests, where high-impact logging cycles occurred; bauxite mining areas, where vegetation and soil were completely removed; areas of abandoned pasture; and post-mining forest regeneration areas that are part of the PRAD (Fig 1). In this study, we sampled three habitats: 1) degraded mature forests, 2) abandoned pasture, and 3) post-mining forest regeneration sites implemented from 2009 to 2012. The area is also surrounded by productive areas, including livestock and monoculture of soybeans and corn, as well as burnt forest patches. There are no areas of mature forest totally preserved in the study region. The hunting activity is discouraged by the Hydro Paragominas Company, however, it is possible to see hunter records in the region [30]. In this work, we considered the hunting activity as a constant variable in all studied habitats.

### Data collection

Field trips for data collection occurred between June 2014 and July 2016. We used 35 camera traps [31] to record the four target species of this study. We spread the camera-traps throughout the study area to sample the maximum of its environmental variability (Fig 1). We consider a grid of 3 x 3 km implemented on a satellite image of the area and installed the cameras as close as possible to the coordinates of the vertices of this grid. Some vertices were too difficult to reach, and we placed the cameras as close as possible. As we didn´t collect or transport any biological material, and the study wasn´t developed in an official Protected Area, we didn´t need special authorization from the Brazilian government to collect the information on the field. We installed all the camera-traps at a height of approximately 40 cm from the ground and left them running uninterrupted throughout the duration of the study. We checked the camera-traps every 90 to 120 days, to change SD-cards with photos, to exchange batteries or replace cameras when necessary. We programmed the camera-traps to take 3 photos every 30 seconds, recording the date and time of each record, as well as the geographical coordinates of the place. We consider each trap as a sampling unit. A camera-trap photograph was defined as an independent event if consecutive photos recorded (i) one or more individuals of different species; or (ii) one or more individuals of the same species over a minimum time interval greater than 60 min [32, 33, 34]. Using these criteria, all photos defined as non-independent were excluded from subsequent analyses. We used the program Camera Base version 1.7 (http://www.atrium-biodiversity.org/tools/camerabase/) to process and store the photo records from the camera-traps.

### Sampling of environmental variables

We measured environmental and anthropogenic variables to verify their influence on the abundance of mammalian species. We used a protocol adapted from Gonçalves et al. [35] and based on the work of Peck et al. [36], which evaluates habitat characteristics and human influence. At all camera traps we placed two plots of 50 m x 10 m, located at each side of the camera trap where we measured some of the environmental variables. For each camera-trap we recorded 21 variables that could be related to the species occurrences: 1) Proportion of the area covered by water, 2) Proportion of deforestation area, 3) Proportion of degraded mature forest, 4) Proportion of riparian area, 5) Proportion of regeneration area, 6) Estimated number of seedlings in plot, 7) Distance from degraded mature forest (m), 8) Depth of litter, 9) Number of standing dead trees, 10) Number of fallen dead trees, 11) Proportion of trees with DAP < 55 cm, 12) Proportion of trees with DAP > 55 cm, 13) Canopy height, 14) Proportion of trees with lianas, 15) Average canopy opening, 16) Distance to permanent watercourse, 17) Distance to productive area, 18) Distance to burned area, 19) Sub-surface opening ratio, 20) Distance to mining area, and 21) Minimum distance to trail /road (S1 Table). We collected the variables 6, 8, 9, 10, 11, 12, 13, 14, 15 and 19 at the fieldwork using the plots of 50 m x 10 m, located at each side of the camera trap., while the other variables were collected using satellite images, available at the site of the *Instituto do Homem e Meio Ambiente da Amazônia*–IMAZON (https://imazon.org.br) (S1 Table). We used ArcGis version 10.2 software and the classified shapefile from the study area, to extract the vegetation and land-use variables 1, 2, 3, 4, 5. 7, 20 and 21. We designed the buffers of 1 km around each camera trap to calculate the proportion of all kind of vegetation cover and water surface using the ArcGis [37]. The size of the buffer was based on the independence of the sampling of the variables. We also measured the perpendicular distance from each sample unit to the nearest forested area (DF), to the nearest permanent water body (DW), and to the nearest mining area (DM), using the ArcGis.

To characterize the habitat structure, we calculated the percentage of canopy opening (CO) in each camera trap sampling point. We took five photos for sampling point, one at each 50 m x 10 m sampling plot and one right where the camera trap was positioned. We used a camera with a fisheye lens, positioned 1.20 m from the ground, fully directed to the canopy. The photos were analyzed in the software ENVI 5.3, where we calculated the average percentage of canopy opening (AD) for each sampling point similar to that proposed by Marsden et al. [38] for sub-forest complexity analysis [38].

Using a Principal Component Analysis (PCA) we selected 5 environmental variables including: proportion of degraded mature forest (MF), canopy opening (CO), distance from the degraded mature forest (DF), distance from permanent watercourse (DW) and distance to the mining area (DM).

### Data analysis

We used PCA to select some of the correlated environmental variables and avoid multicollinearity. Our selection criterion is based on the main variables of each ordination axes that indicate the most influential variables to our analyzed species. This analysis provided the most important information between the 21 variables sampled (S1 Table), and we used the broken stick criterion as complementary analysis to determine the most important variables [39]. We use the R platform through the vegan [40], permute [41], lattice [42] and MASS [43] packets to perform the analyzes.

We used Generalized Linear Mixed Models (GLMM) [44] to evaluate the influence of the predictor variables on the abundance of the species. In this case, we used the numbers of days of exposure of each camera trap as a random effect and the 5 selected environmental variables as fixed effects. We used the Poisson distribution family (log linking function) since the data residues did not fit to the Gaussian distribution family. To analyze all the possible effects of the predictive variables isolated and the combinations of these variables, we built different models considering all possible combinations between the predictor variables (S2 Table). We used the BOBYQA optimizer to obtain the best performance in the convergence analysis [45]. To select the best model, we used the Akaike Information Criterion adjusted for small samples (AICc) [46]. For these analyses, we used the AICcmodavg package [47], which makes the selection of the most parsimonious model. The model with the lowest AICc value and ΔAICc lesser than 2 was considered the model with the best fit [46]. To generate the GLMMs we use the glmer function, present in the lme4 package [48] and bbmle package [49]. For the calculation of the pseud R^2^ partial and conditional [50], we used the MuMin package [51] (S3 Table). All the analyses were done in Software R 3.4.1 [52]. In the S2 Table are predictor variables included in each model of GLMM analysis.

For more descriptive analyses between habitats, we used the Abundance Rate, calculated considering individual species records as independent photographic records per 100 functioning camera-trap night (FCTNs). The mean FCTNs per camera trap deployment was 572.34 ± 161.42. We compared the abundance between habitats observing the overlap of the confidence interval of the averages. To understand the relationship between the habitats and the environmental variables, we used the PCA [53].

## Results

We obtained 2059 independent records of the four endangered species evaluated in this study, of which 263 were of *M*. *tridactyla*, 50 of *P*. *maximus*, 1585 of *T*. *terrestris* and 161 of *T*. *pecari*. All four species were widely distributed in the study area.

**Fig 2:**
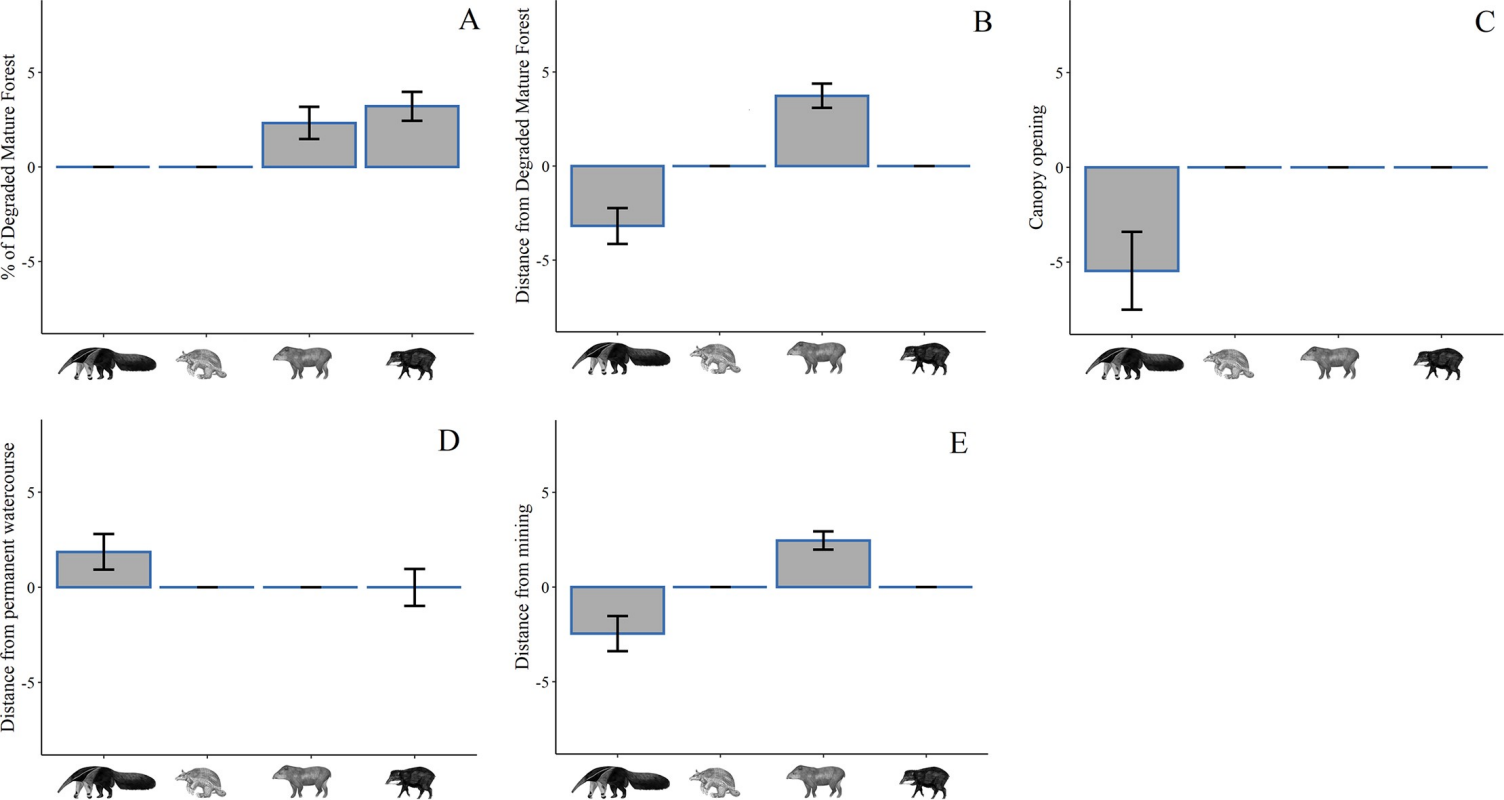
Predictor variables selected by the mixed generalized linear models and the size of the effect for each species. Detail of the effect size and the influence, positive or negative, of each variable for each species. Variables analyzed: (A) Proportion of degraded mature forest (MF), (B) Distance from the degraded mature forest (DF), (C) Average canopy opening (CO), (D) Distance from permanent watercourses (DW), (E) Distance from the mining (DM). Species analyzed from left to right in the X axes of each plot: *M*. *tridactyla* (Giant anteater); *P*. *maximus* (Giant armadillo); *T*. *terrestris* (Lowland tapir); *T*. *pecari* (White-lipped peccary).

For the species, *M*. *tridactyla*, the global model considering all the predictive variables (ΔAICc = 0.00), was the most adequate to explain the variation of the abundance of this species (S3 Table). When assessing the relative importance of each variable alone, only MF does not affect the abundance rate of this species (Fig 2, Table 1). The DF, CO, and DM have a negative influence on the abundance rate of *M*. *tridactyla* (Table 1), indicating that they prefer areas not distant from the mining but also not distant from the forest. On the other hand, the greater the DW, the higher the abundance rate of *M*. *tridactyla*.

**Table 1:** Results for predictor variables selected by the GLMM. Models for *M*. *tridactyla* (Giant anteater), *P*. *maximus* (Giant armadillo), *T*. *terrestris* (Lowland tapir) and *T*. *pecari* (White-lipped peccary).

Species		Predictor variables	*β*	SE	z-test	P
*M*. *tridactyla*		Proportion of degraded mature forest (MF)	-1.729	1.119	-1.545	0.122
		Distance from the degraded mature forest (DF)	**-3.178**	**0.953**	**-3.334**	**< 0.001**
		Average canopy opening (CO)	**-5.453**	**2.053**	**-2.657**	**0.008**
		Distance from watercourse (DW)	**1.862**	**0.932**	**1.999**	**0.046**
		Distance from the mining (DM)	**-2.460**	**0.927**	**-2.654**	**0.008**
*P*. *maximus*		Average canopy opening (CO)	-3.070	1.865	-1.646	0.100
*T*. *terrestris*		Proportion of degraded mature forest (MF)	**2.326**	**0.8445**	**2.754**	**< 0.001**
		Distance from the degraded mature forest (DF)	**3.731**	**0.644**	**5.793**	**< 0.001**
		Average canopy opening (CO)	2.198	1.265	1.738	0.082
		Distance from watercourse (DW)	-1.119	0.656	-1.707	0.088
		Distance from the mining (DM)	**2.452**	**0.486**	**5.045**	**< 0.001**
*T*. *pecari*	Model5	Proportion of degraded mature forest (MF)	3.207	0.766	4.188	**< 0.001**
*T*. *pecari*	Model2	Proportion of degraded mature forest (MF)	3.987	1.252	3.184	**< 0.001**
		Distance from the degraded mature forest (DF)	0.360	1.500	0.240	0.810
		Average canopy opening (CO)	-0.751	1.428	-0.526	0.599
		Distance from watercourse (DW)	-2.572	0.974	-2.641	**< 0.001**

Bold values indicate interactions at the level of significance of P < 0.05.

For *P*. *maximus* the most suitable model to explain the variation in abundance included only CO (ΔAICc = 0.00). However, this variable had no significant effect (Table 1 and S3 Table). The second model selected was the null model, so we only considered the information from the first model for discussion. For *T*. *terrestris*, the global model considering all the predictive variables (ΔAICc = 0.00), was the most adequate model to explain the variation in abundance rate for this species (S3 Table). We observed that MF, DF, CO and DM increase the abundance (Fig 2), while DW decreases the abundance of this species (Table 1, Fig 2D). The CO and the DW were not individually significant but, together with the other variables influenced the abundance of *T*. *terrestris* (Table 1, Fig 2C and Fig 2D). For *T*. *pecari* only the model including the MF (ΔAICc = 0.00), was the most adequate to explain the variation of the abundance (S3 Table). In this model, the MF was significant and had a positive influence on the abundance of *T*. *pecari* (Table 1, Fig 2A). However, in the second selected model, we observed that DW presented as a significant variable (Table 1, Fig 2D).

The PCA results showed that the environmental variables MF, DW and DM are positively related to the habitat of Degraded Mature Forest, while the samples of abandoned pasture and regeneration are more related to the CO and DF (S1 Fig).

**Fig 3:**
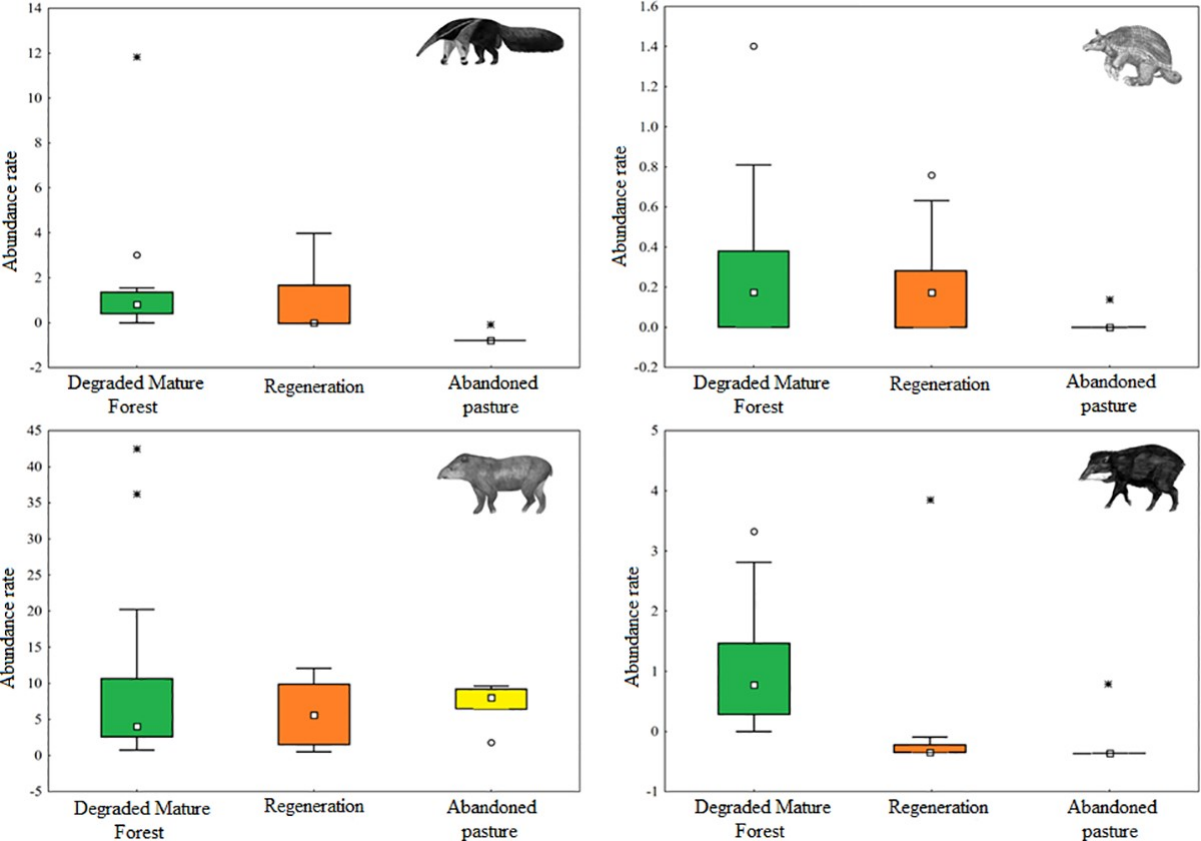
Comparison of the abundance rate averages between habitats and its confidence intervals (95% of confidence). (A) *M*. *tridactyla* (Giant anteater), (B) *P*. *maximus* (Giant armadillo), (C) *T*. *terrestris* (Lowland tapir) and (D) *T*. *pecari* (White-lipped peccary).

**Fig 4:**
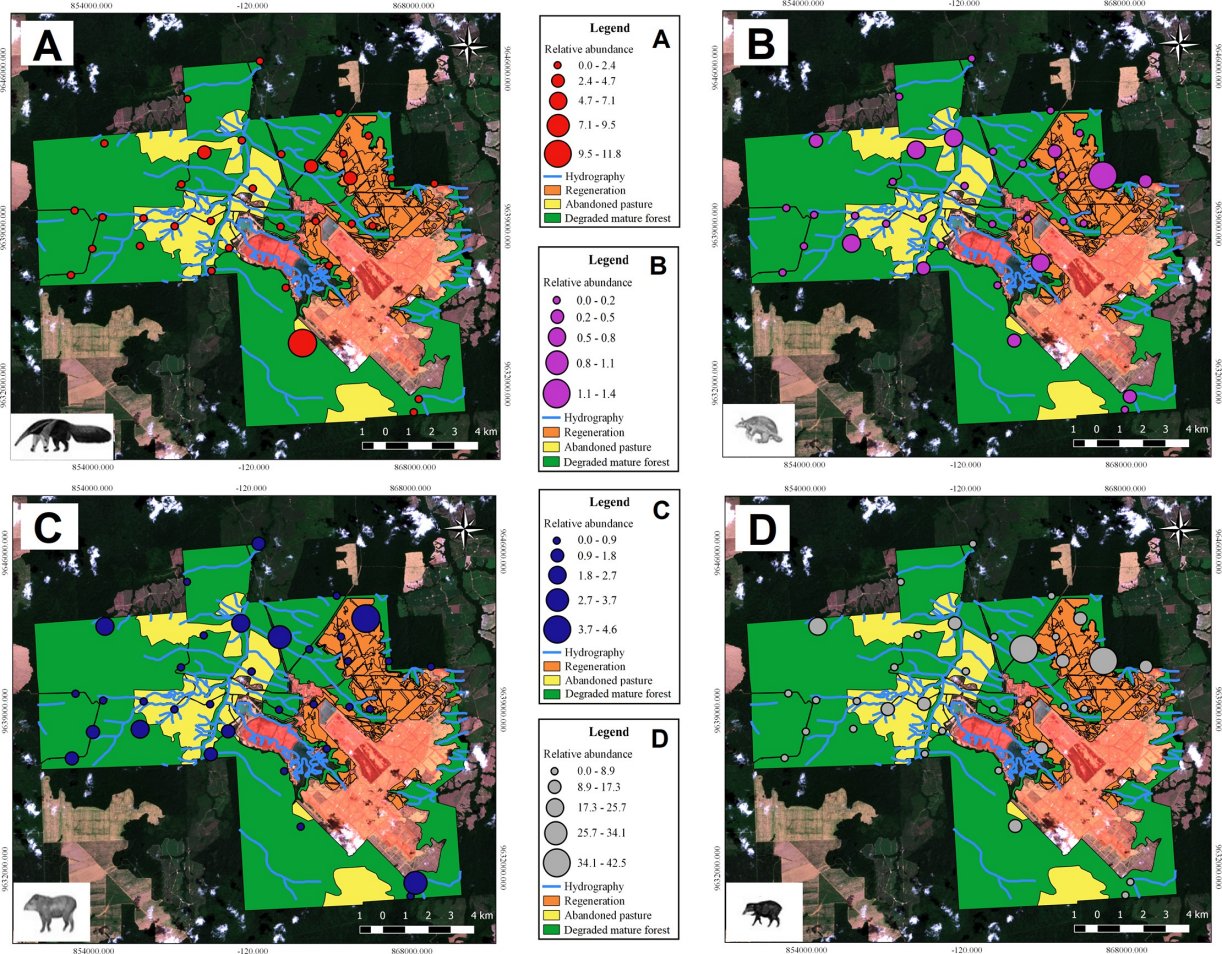
Distribution of each species in the sampled habitat types. (A) *M*. *tridactyla* (Giant anteater), (B) *P*. *maximus* (Giant armadillo), (C) *T*. *terrestris* (Lowland tapir) and (D) *T*. *pecari* (White-lipped peccary). Reprinted from [28] [29] under a CC BY license, with permission from [IMAZON and EOS], original copyright [2013].

The species *M*. *tridactyla* and *P*. *maximus* seems to avoid the abandoned pasture (Fig 3A and 3B, Fig 4A and 4B), but this is more evident in *M*. *tridactyla* (Fig 3A). However, the place where the abundance of *P*. *maximus* was highest in the PRAD areas is positioned at the edge of a plateau, where the area presents a large slope (Fig 4B). In the case of *T*. *terrestris*, we recorded a high abundance of this species in the whole area, especially in the regeneration areas, but also at the degraded mature forest (Fig 4C). There is no difference on abundance rate of *T*. *terrestris* between habitats (Fig 3C). The abandoned pasture seems to be the less used habitat by the four species studied. Considering the three sampled habitats, in general the species had similar preference for the forested environments and for the regeneration areas, except for *T*. *pecari*, that was scarcely recorded outside the forested areas (Figs 3D and 4D).

## Discussion

All four species studied were distributed in all three sampled habitats which suggest that they have some level of tolerance to degradation. However, we observed that the abundance rate of each species in the various habitats was not the same, and the environment variables act in distinct ways on them. This probably will influence the adaptive plasticity of each one different in the long term [11, 13].

The *M*. *tridactyla* was influenced negatively by the DF, DM and CO. This suggests the preference of this species for the edge habitats, that can be defined in this study as open areas, not distant from the mining, but also not distant from the forest. In this case, these characteristics define the recovering habitats. The *M*. *tridactyla* also seems to avoid the abandoned pasture. This species is often found in forested environments with low density of understory, probably due to its locomotion patterns [54]. Individuals of this species showed behavioral changes in areas of high anthropic pressure, becoming more active at night [55]. However, another suggested factor for this behavior change was the increase in temperature [56]. High insolation and humidity causing high temperatures in the Amazon region [57] may be crucial for some species in open anthropogenic areas, even for species considered typical in naturally open areas like the Brazilian Cerrado such as *M*. *tridactyla* [56, 58]. The *M*. *tridactyla* is one of the largest ant and termite eaters in the world [59] and because of its restricted and low-calorie diet, the species has a slow metabolism and has difficulty regulating body temperature itself [56]. The covered habitat can be used as thermic refuge for giant anteaters, to avoid exposure during the hottest hours of the day, but also, they can use more open areas to avoid the coldest hours of the day and for foraging [58]. However, the number of trees and shrubs in an area can influence the habitat preference of this anteater species. In this study, *M*. *tridactyla* had preference both for forest environments, as well as by more open areas, here represented by the regeneration areas. It seemed to avoid the more open areas as abandoned pasture, probably because of the lack of the Shrubbery and the high temperatures [60].

In our study the distribution of *P*. *maximus* was not influenced by any of the measured environmental variables. However, it had a greater abundance in regeneration areas as in degraded mature forest, and less abundance in open areas as abandoned pasture. The *P*. *maximus* seems to avoid areas with dense understory, as a strategy to facilitate its movement [61]. The *P*. *maximus* has a diet of ants and termites similar to *M*. *tridactyla*, but can also feed on other arthropods, carrion and plant material [62, 63]. In our study, the sites with the highest number of records of *P*. *maximus* coincide with the boundary of the bauxite mine plateau area, where the slope of the terrain increases considerably. This species digs burrows in the ground to protect itself from predators and destroys termite and ant mounds to feed [64]. In order to decrease the energy, cost of digging they prefer to make burrows in sloped terrain [65]. The species is considered naturally rare in nature [66] and in our study was the species with the lowest number of records.

The *T*. *terrestris* was the species with the highest abundance in all three habitats sampled, indicating that it probably has the greatest ecological plasticity between all four species studied. The large felids *Panthera onca* and *Puma concolor* are distributed in the study area but the tapir seems not to be the preferred prey of these species, due to the high cost of hunting [67, 68]. In general, hunting by humans may be the greatest threat to tapirs in the Amazon [69, 70]. The hunting activity in the study area seems to be more sportive, practiced with the use of dogs to select some target species, especially deers (*Mazama americana* and *Mazama nemorivaga*) and pacas (*Cuniculus paca*) [30]. The lack of predation and hunting and a high abundance of food resources, especially in the regeneration areas, may be the main causes of the high rate of tapirs recorded in the study area [71,72].

The *T*. *terrestris* was positively influenced by the environmental variables tested, except the DW, which had a negative influence. This species is known to be highly dependent on aquatic environments for regulation of the intestinal tract, thermoregulation, elimination of ectoparasites, and as shelter against predators [73,74]. In this study we observed a preference for regeneration areas, probably due to high abundance of food resources in these areas. *T*. *terrestris* is the largest herbivore in South America and feed daily on huge quantities of fallen fruits, leaves, stems and sprouts. Due to the low efficiency of its digestive system for cellulose fermentation, this animal spends a great part of its day feeding [75].

Among the four species studied, *T*. *pecari* seems to be the one with the least preference for degraded environments. The only variable that positively influenced the relative abundance of this species was the MF. Although *T*. *pecari* is considered omnivorous, feeding on seeds, invertebrates, small vertebrates and larger carcasses, this species prefers a frugivorous diet [76]. This type of diet normally is dependent on a high-quality habitat [77]. *T*. *pecari* usually lives in large social groups, ranging from 10 to 300 individuals, but depending on the environmental conditions [78]. Due to a great bite force, these animals are able to feed on hard fruits and beans with medium seeds, about 1–3 cm, which are more common in mature forests than in regeneration areas [79, 80].

The environmental changes occurring in the study area due to the bauxite mining fit the concept of HIREC suggested by Sih et al. [12]. HIREC may alter interspecific and intraspecific interactions, leading to reduced species richness, behavioral changes, or spatiotemporal conditions [12, 81, 82, 83]. These changes may favor new evolutionary responses to HIREC in the long term [84, 85]. The study area has been undergoing profound changes in its vegetation cover, with several economic cycles occurring in the last 60 years. These changes can be considered to have led to "novel" or "emerging" ecosystems [12, 13], to which the terrestrial Amazonian mammal fauna is adapting. However, taking our results as examples of the "ecological trap" phenomenon [13, 14] may be premature since we did not measure the fitness changes of the species over time. But the positive influence of anthropogenically altered habitats on species abundances in this study can be considered as a potential indication of this phenomenon. In this case, regeneration areas could be considered "ecological trap" [13, 14] for at least three of the four species studied, *M*. *tridactyla*, *P*. *maximus*, *and T*. *terrestris*.

In spite of the tolerance of the species studied to the degraded habitats and the ability to occupy regeneration areas, with the exception of *P*. *maximus*, the distribution of the other species *M*. *tridactyla*, *T*. *terrestris* and *T*. *pecari* were all positively influenced by forested environments. We observed that the occurrence of the species in the degraded areas depends on the presence of the forested areas. This study reinforces that, in regions of high anthropogenic pressure, as is the case in the eastern Brazilian Amazon, all forest remnants, whether degraded or secondary, at different levels of degradation, are important for the survival of endangered mammal species [10, 86, 87].

The rapid changes that the Amazon rainforest has been undergoing, mainly in its agricultural frontier areas due to the advancement of anthropic pressures, have put the fauna in a fragile position and caught in an “Ecological Trap”. Less susceptible to these changes are those organisms that evolutionarily went through similar situations [13]. Many medium and large-sized mammal species are still present in the degraded areas of eastern Amazonia [8, 10]. However, how long will these species survive in these degraded forest remnants? Which species will be able to sustain themselves in the long term, and will the future diversity be high enough to keep the ecosystem functioning? These are important questions that demand detailed long term population studies correlating with the environmental variables of the different habitats, like the present research.

It is quite clear to us that forest remnants are relevant to the conservation of fauna in the eastern Brazilian Amazon, but the presence of large protected areas would be much better to meet the ecological demands of larger mammals. However, the presence of these extensive conserved forest areas is already severely compromised in this eastern Amazon region [1, 2]. Another alternative has been to invest in the forest recovery in degraded areas [88]. The problem is that the recovery of the forest seems to be much slower than the rate of the forest destruction. Thus, focusing on studies and efforts to increase the efficiency of the restoration of degraded areas, which may even serve as corridors between the forest remnants, may be one of the strategies to favor the conservation of the fauna in eastern Brazilian Amazon.

## Supporting information

S1 Fig PCA results showing that the environmental variables MF, DW and DM are positively related to the habitat of degraded mature forest, while the samples of abandoned pasture and regeneration are more related to the CO and DF. (DOCX)

S1 Table Environmental and anthropogenic variables measured in the field or by satellite images at the sampling points, showing the minimum and maximum values. (DOCX)

S2 Table Predictor variables included in each model of GLMM analysis. (DOCX)

S3 Table Results of model selection by AICc at GLMM analysis. (DOCX)

S4 Table Number of records of each species in each sample unit. (XLSX)
