# Supplementary material for: Environmental factors influencing the abundance of four species of threatened mammals in degraded habitats in the eastern Brazilian Amazon
Source: PLoS One. 2020 Feb 26;15(2):e0229459. doi: 10.1371/journal.pone.0229459 (PMC7043734; doi:10.1371/journal.pone.0229459)

**S4 Fig** - PCA results showing that the environmental variables MF, DW and DM are positively related to the habitat of degraded mature forest (green), while the samples of abandoned pasture (yellow) and regeneration (red) are more related to the CO and DF.
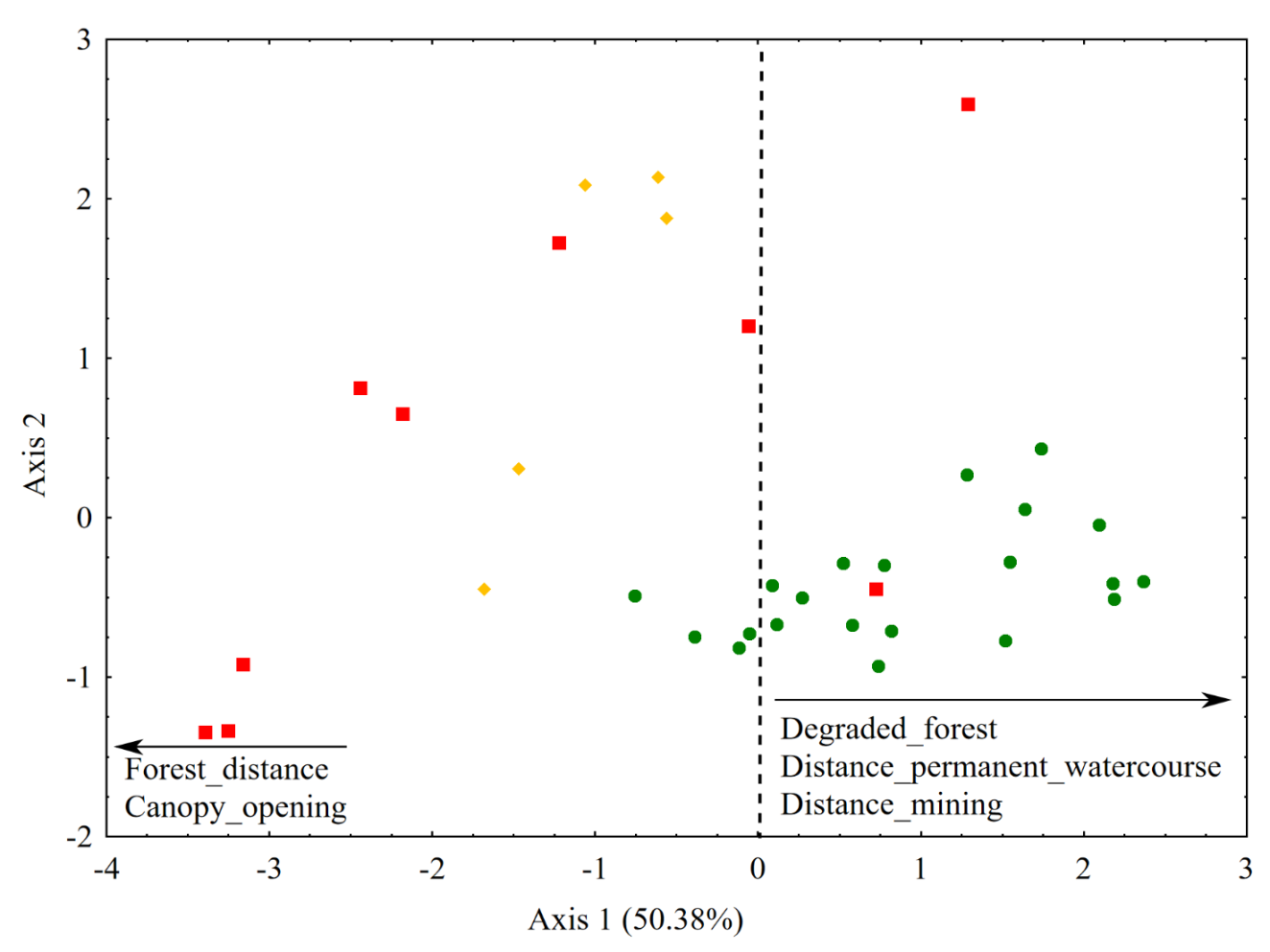

Supplement: S1 Fig — (DOCX) [file pone.0229459.s001.docx]
