# Supplementary material for: Environmental factors influencing the abundance of four species of threatened mammals in degraded habitats in the eastern Brazilian Amazon
Source: PLoS One. 2020 Feb 26;15(2):e0229459. doi: 10.1371/journal.pone.0229459 (PMC7043734; doi:10.1371/journal.pone.0229459)
Supplement: S1 Table — (DOCX) [file pone.0229459.s002.docx]

**S1 Table** - Environmental and anthropogenic variables measured in the field or by satellite images at the sampling points, showing the minimum and maximum values.

|  | Variables | Chosen | Minimum | Maximum |
| --- | --- | --- | --- | --- |
| 1 | Proportion of the area covered by water | No | 0 | 0.19 |
| 2 | Proportion of deforestation area | No | 0 | 3.0 |
| 3 | Proportion of degraded mature forest (MF) | Yes | 0 | 3.14 |
| 4 | Proportion of riparian area | No | 0 | 0.06 |
| 5 | Proportion of regeneration area | No | 0 | 2.36 |
| 6 | Estimated number of seedlings in plot | No | 0 | 62 |
| 7 | Distance from degraded primary forest (m) (DF) | Yes | 0 | 1695 |
| 8 | Depth of litter (cm) | No | 0.2 | 7.8 |
| 9 | Number of standing dead trees | No | 1 | 49 |
| 10 | Number of fallen dead trees | No | 0 | 42 |
| 11 | Proportion of trees with DAP < 55 cm | No | 86.2 | 100 |
| 12 | Proportion of trees with DAP > 55 cm | No | 0 | 13.8 |
| 13 | Canopy height (m) | No | 3 | 34.2 |
| 14 | Proportion of trees with lianas | Yes | 18.03 | 96.2 |
| 15 | Average canopy opening (AD) | Yes | 24.1 | 82.3 |
| 16 | Distance to permanent watercourse (m) (DA) | Yes | 8 | 3770 |
| 17 | Distance to productive area (m) (DP) | Yes | 10 | 4050 |
| 18 | Distance to burned area (m) | Yes | 335 | 11850 |
| 19 | Sub-surface opening ratio | No | 41.7 | 71.5 |
| 20 | Distance to mining area (m) | Yes | 231 | 8045 |
| 21 | Minimum distance to trail/road (m) | No | 13 | 1500 |
