# Supplementary material for: Environmental factors influencing the abundance of four species of threatened mammals in degraded habitats in the eastern Brazilian Amazon
Source: PLoS One. 2020 Feb 26;15(2):e0229459. doi: 10.1371/journal.pone.0229459 (PMC7043734; doi:10.1371/journal.pone.0229459)
Supplement: S2 Table — (DOCX) [file pone.0229459.s003.docx]

**S2 Table** - Predictor variables included in each model of GLMM analysis.

| Models | Predictor variables |
| --- | --- |
| Model 1 | PF+DF+CO+DW+DM |
| Model 2 | PF+DF+CO+DW |
| Model 3 | PF+DF+CO |
| Model 4 | PF+DF |
| Model 5 | PF |
| Model 6 | DF |
| Model 7 | CO |
| Model 8 | DW |
| Model 9 | DM |
