# Supplementary material for: Environmental factors influencing the abundance of four species of threatened mammals in degraded habitats in the eastern Brazilian Amazon
Source: PLoS One. 2020 Feb 26;15(2):e0229459. doi: 10.1371/journal.pone.0229459 (PMC7043734; doi:10.1371/journal.pone.0229459)
Supplement: S3 Table — (DOCX) [file pone.0229459.s004.docx]

**S3 Table** - Results of model selection by AICc at GLMM analysis. PseudoR² parcial= R^2^m and PseudoR² conditional= R^2^c.

| Model | *M. tridactyla* | | | Model | *P. maximus* | | | Model | *T. terrestris* | | | Model | *T. pecari* | | |
| --- | --- | --- | --- | --- | --- | --- | --- | --- | --- | --- | --- | --- | --- | --- | --- |
|  | AICc | dAICc | df |  | AICc | dAICc | df |  | AICc | dAICc | df |  | AICc | dAICc | df |
| Model 1  (R^2^m=0.321,  R^2^c= 0.689) | 211.4 | 0.0 | 7 | **Model 7**  (R^2^m=0.152, R^2^c= 0.619) | 107.9 | 0.0 | 3 | **Model 1**  (R^2^m=0.195, R^2^c= 0.382) | 352.6 | 0.0 | 7 | **Model 5**  (R^2^m= 0.304, R^2^c=0.665) | 180.4 | 0.0 | 3 |
| Model 2 | 215.8 | 4.4 | 6 | **Model 0** | 108.6 | 0.7 | 2 | **Model 2** | 379.5 | 26.9 | 6 | **Model 2** | 180.5 | 0.1 | 6 |
| Model 3 | 220.2 | 8.9 | 5 | **Model 9** | 110.1 | 2.2 | 3 | **Model 3** | 394.1 | 41.5 | 5 | **Model 4** | 182.8 | 2.4 | 4 |
| Model 8 | 221.1 | 9.7 | 3 | **Model 8** | 110.8 | 2.9 | 3 | **Model 4** | 400.0 | 47.4 | 4 | **Model 1** | 183.7 | 3.3 | 7 |
| Model 4 | 227.0 | 15.6 | 4 | **Model 5** | 110.9 | 3.0 | 3 | **Model 9** | 435.3 | 82.7 | 3 | **Model 3** | 185.0 | 4.6 | 5 |
| Model 7 | 227.3 | 15.9 | 3 | **Model 6** | 110.9 | 3.0 | 3 | **Model 8** | 449.9 | 97.3 | 3 | **Model 9** | 191.1 | 10.7 | 3 |
| Model 6 | 228.4 | 17.0 | 3 | **Model 3** | 112.7 | 4.8 | 5 | **Model 5** | 465.2 | 112.6 | 3 | **Model 6** | 191.3 | 10.9 | 3 |
| Model 0 | 228.7 | 17.4 | 2 | **Model 4** | 113.5 | 5.6 | 4 | **Model 0** | 468.4 | 115.8 | 2 | **Model 0** | 207.7 | 27.2 | 2 |
| Model 9 | 230.0 | 18.7 | 3 | **Model 1** | 115.2 | 7.3 | 7 | **Model 7** | 470.2 | 117.6 | 3 | **Model 7** | 209.0 | 28.6 | 3 |
| Model 5 | 230.6 | 19.2 | 3 | **Model 2** | 115.4 | 7.5 | 6 | **Model 6** | 470.8 | 118.2 | 3 | **Model 8** | 209.8 | 29.4 | 3 |
